# Simulation and Modeling of Telocytes Behavior in Signaling and Intercellular Communication Processes

**DOI:** 10.3390/ijms21072615

**Published:** 2020-04-09

**Authors:** Dragos Cretoiu, Simona Roatesi, Ion Bica, Cezar Plesca, Amado Stefan, Oana Bajenaru, Carmen Elena Condrat, Sanda Maria Cretoiu

**Affiliations:** 1 Department of Cell and Molecular Biology and Histology, Carol Davila University of Medicine and Pharmacy, 050474 Bucharest, Romania; dragos@cretoiu.ro (D.C.); sanda@cretoiu.ro (S.M.C.); 2 Alessandrescu-Rusescu National Institute of Mother and Child Health, Fetal Medicine Excellence Research Center, 020395 Bucharest, Romania; drcarmencondrat@gmail.com; 3 Department of Applied Informatics, Faculty of Information Systems and Cyber Security, “Ferdinand I” Military Technical Academy, 050141 Bucharest, Romania; cezar.plesca@mta.ro; 4 Department of Computers and Cyber Security, Faculty of Information Systems and Cyber Security, “Ferdinand I” Military Technical Academy, 050141 Bucharest, Romania; ion.bica@mta.ro; 5 Department of Integrated Systems of Aviation and Mechanics, Faculty of Aircraft and Military Vehicles, Technical Military Academy “Ferdinand I”, 050141 Bucharest, Romania; amado.stefan@mta.ro; 6 Crescendo International, 020103 Bucharest, Romania; oana.bajenaru@crescendo.ro

**Keywords:** telocytes, telocyte network tracking and indexing, content-based image retrieval, image indexation, modeling of living cell behavior, viscoelastic constitutive laws

## Abstract

Background: Telocytes (TCs) are unique interstitial or stromal cells of mesodermal origin, defined by long cellular extensions called telopodes (Tps) which form a network, connecting them to surrounding cells. TCs were previously found around stem and progenitor cells, and were thought to be most likely involved in local tissue metabolic equilibrium and regeneration. The roles of telocytes are still under scientific scrutiny, with existing studies suggesting they possess various functions depending on their location. Methods: Human myometrium biopsies were collected from pregnant and non-pregnant women, telocytes were then investigated in myometrial interstitial cell cultures based on morphological criteria and later prepared for time-lapse microscopy. Semi-analytical and numerical solutions were developed to highlight the geometric characteristics and the behavior of telocytes. Results: Results were gathered in a database which would further allow efficient telocyte tracking and indexing in a content-based image retrieval (CBIR) of digital medical images. Mathematical analysis revealed pivotal information regarding the homogeneity, hardness and resistance of telocytes’ structure. Cellular activity models were monitored in vitro, therefore supporting the creation of databases of telocyte images. Conclusions: The obtained images were analyzed, using segmentation techniques and mathematical models in conjunction with computer simulation, in order to depict TCs behavior in relation to surrounding cells. This paper brings an important contribution to the development of bioinformatics systems by creating software-based telocyte models that could be used both for diagnostic and educational purposes.

## 1. Introduction

Biological structures are very intricate systems with a three-dimensional organization that is quite challenging to recreate in in vitro models [1]. Although progress in this regard is being made, we are still unable to fully reproduce the complexity of living organisms. One of the major targets of biological systems researchers is being able to successfully transpose research models into living bodies or tissues/cells. The interest in understanding cellular morphology dates back to the early 1960s, when numerous reconstruction techniques were attempted, many focusing on the three-dimensional structure of neurons [2,3]. Generally, in order to get a better grasp on tissue morphology, appropriate visual models are needed. Recent upgrading of computers as well as advanced digital processing technology greatly improves the three-dimensional reconstruction of biological structures [4,5].

Over the last decade, a lot of information has been gathered regarding the existence of telocytes (TCs) in both cavitary and parenchymatous organs, in humans and other mammals [6,7,8,9,10,11,12,13,14,15]. The newly introduced term, “telocyte” (TC), replaces the previously used name, “interstitial Cajal-like cell” (ICLC), and it is meant to avoid confusion with other types of interstitial cells, such as interstitial cells of Cajal (ICC)—for extensive reviews see [16,17,18,19,20].

Telocytes are mainly defined by their ultrastructural features, which are marked by the presence of very long extensions that reach between tens and hundreds of micrometers, called telopodes (Tps) [21,22]. TCs have small bodies and a variable number of Tps. Depending on the number of Tps, TCs can have a piriform, spindle, triangular or stellate shape [23]. The nucleus is oval, with moderately dense chromatin and no obvious nucleolus. The cytoplasm surrounding the nucleus is scarce and contains a small Golgi apparatus, some mitochondria, and few cisternae of rough and smooth endoplasmic reticulum [24]. Telopodes, ranging from one to five per cell, are connected to each other through atypical junctions that form an interstitial network. Telopodes have a moniliform aspect due to the alternation of podomers, defined as thin segments that usually measure less than 0.2 μm, below the resolving power of light microscopy, and podoms, which are dilated segments accommodating mitochondria, endoplasmic reticulum and caveolae [9,16]. Through their Tps, TCs release exosomes, which are vesicles ranging from 60 to 100 nm in diameter, as well as clusters of shedding microvesicles (SMVs), with diameters between 250 and 350 nm and 1 mm [25].

Some of the most intriguing hypotheses regarding the roles of TCs refer to long distance intercellular communication and organ specific renewal [25]. Telocytes are believed to be involved in intercellular communication either with the aid of the junctions that they set up, or through the release of microvesicles [26]. This way, they establish complex networks that sustain the physiological organ-wide coordination of heterocellular signaling. They are also thought to play a significant role in organ renewal due to their presence in stem cell niches. Telocytes could act as stromal support cells for stem cells in various organs, e.g., heart, lung, striated muscle, integrated in the concept of “Telocytes/Stem-Cells Tandem” [27,28].

It is presently recognized that cells can change their shape, structure and mechanical properties in response to mechanical stress [29]. Since TCs are such versatile cells, their cytoskeletal structure actively and closely influences their function, especially their role in intercellular communication in a given environment [30]. Therefore, it is important to determine the constitutive properties of these cells and to generate a quantitative description of the cellular mechanics and dynamics in order to further assess the signaling processes involved in intercellular communication and tissue regeneration. This issue is achieved through both thorough and systematic laboratory tests and appropriate numerical simulations. Understanding the mechanisms by which cells respond to changes in the surrounding matrix is crucial in regenerative medicine in general and in influencing cellular differentiation in particular. Moreover, changes in constitutive properties may contribute to the development of various pathologies.

Cellular intercommunication is a complex process and its analysis through common imaging techniques such as light microscopy, electron microscopy and immunohistochemistry, has important limitations. Progress can be made in understanding TCs’ behavior, morphology and signaling mechanisms by constructing and analyzing abstract analogues in software. In this way, features can be modeled and explored at all levels. We believe that scientifically useful mappings represent a bridge between digital modeling and in vitro cell cultures. Though in vitro telocyte cultures offer a much simpler representation of cells than actual mammalian tissues, they still provide an adequate level of complexity [4,31]. The techniques used in this paper include theoretical modeling and numerical simulations regarding telocyte behavior and their potential role in regenerative medicine, taking into account existing literature data on their biological, chemical, electrical and mechanical characteristics.

However, an important issue is posed by the assembling of the digital content, both images and videos, into a multimedia database that should provide indexation and quick searching facilities in order to find the desired content through text or content-based queries. Nowadays, in the medical field, a large number of imaging techniques are utilized daily (e.g., Computer Tomography, Magnetic Resonance, etc.) in order to aid in clinical decision making. These areas could undergo further development, thus providing valuable teaching, training and enhanced image interpretation support, by cultivating techniques that support the archiving and retrieval of images by content [32]. In this manner, before making a diagnosis, clinicians would be able to retrieve similar cases from the medical archive. Content-based image retrievals would not only show cases with similar image examinations and diagnoses, but also cases with similar image examinations and different diagnoses [33]. The effectiveness of such a system would ultimately depend on the types of images and the correctness of content representation, the types of image queries allowed and the efficiency of implemented search techniques. Query formulation should be flexible and appropriate, as opposed to queries expressed by a command-oriented query language like SQL. It would be ideal that queries be specified through a GUI (Graphical User Interface), for example by providing an example image or by drawing a sketch. Query by example allows even complicated queries: the user may specify several objects with complex shapes and interrelationships, and may ask for all images containing similar objects with similar relationships. The retrieved images do not need to be exactly similar to the query, but the database search has to be approximate, so that all images up to a pre-specified degree of similarity should be retrieved [34].

Some automatic image indexing methods have been proposed, based on shape [35,36], color [37], or both [38]. The general approach is to calculate some approximate invariant statistics, such as a color histogram, invariants of shape moments, parametric curve distance or frequency sub-band decomposition, and use these to categorize the image database. Our methodology for database design used an efficient representation of image content, providing support for queries by example and approximate image retrieval by content.

This paper is therefore aimed at connecting information from fundamental research and bioinformatic systems for modeling and monitoring cellular activity. The final goal is to study the physical characteristics of TCs in situ on electron microscopy images in different tissues and the in vitro development of Tps and their homo- or/and hetero-cellular contacts, depending on the surrounding medium. These results are incorporated in a special software to create predictive multiscale models as a foundation for an integrative, systems-level approach that offers the opportunity to observe TCs’ involvement in intercellular signaling and tissue regeneration and to identify, simulate and potentially optimize therapeutic means.

The conclusions of our study along with the digital source content will also be integrated as e-learning content for distribution and didactic purposes. It will be available in Sharable Content Object Reference Model (SCORM) format for interoperability and will eventually be easily deployed on every e-learning platform available.

## 2. Results

### 2.1. Imaging

Electron microscopy images were obtained from areas where telocytes were identified in order to observe their full length and structure. Between 11 and 15 images were used for each cell, so that we could obtain an accurate bidimensional assessment. Through imaging and video observations, the capacity of telocytes to create three-dimensional labyrinthic cellular networks was analyzed, as well as the establishment of homo- and hetero-typic interactions of telocytes and anchoring junctions with other types of cells, such as fibroblasts, muscle cells, endothelial and/or epithelial cells. These effects were observed on short- (tens of minutes), medium- (tens of hours) and long-term basis (2–3 days). The observed phenomena consisted of the establishment of specific migration pathways along telopodes, which were responsible not only for the activation and initiation of cellular division, but also for the changes in morphology and the functional shift.

### 2.2. Mathematical Modeling

**Figure 1:**
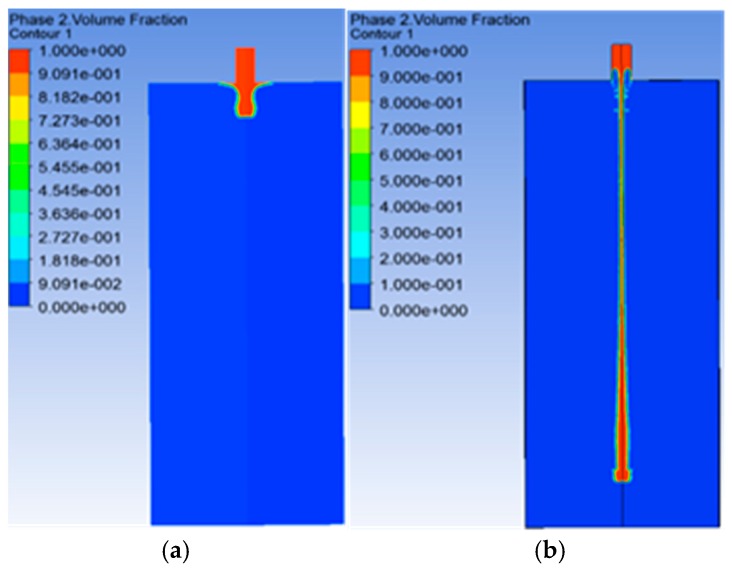
The numerical results of telopode elongation at t = 0 s (**a**) and t = 0.1027 s (**b**), obtained through the finite element method model using a viscoelastic constitutive law for the mathematical model [4]. The numerical model simulates the telopode elongation, the main feature of telocytes (TCs) behavior, and it presents the tracking of the interface between phase 0 (outside the telocyte, marked in blue) and phase 1 (the telocyte body marked in red) by the volume fraction entity.

Figure 1 depicts the numerical results of the telopodes elongation simulation, the main characteristic of TCs behavior. The calculation was performed using the viscoelastic model for the telopode’s elongation, within a multiphase flow as interpenetrating continua. It includes the concept of phasic volume fractions, with values between the phase 0 (outside the telocyte, marked in blue) and phase 1 (the telocyte body, marked in red).

**Figure 2:**
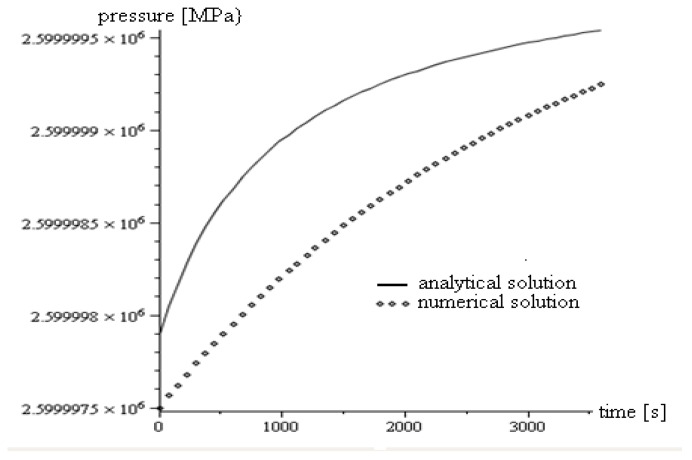
Comparison of semi-analytical and numerical solutions for the same data. The difference between the two types of solutions is due to the inherent limitations of the semi-analytical solution. The numerical solution is able to provide more information regarding the entities involved in the problem considered, while the semi-analytical solution, which is faster, is necessary for validating the numerical calculation [4].

We compared semi-analytical and numerical models using the same material constants in order to validate the results, which we illustrated in Figure 2.

Shape improvement has been one of the most important aspects of CBIR. Shape matching is difficult due to the fact that the human eye may consider shapes similar even when two shapes are non-identically deformed versions of each other. Especially in the medical field, challenges occur in representing both the static and dynamic properties of cell shapes. While a wide array of shape-based description and matching methods have been proposed, few can explicitly accommodate the non-rigid deformations that frequently occur in the types of objects appearing in medical images. For example, in IBM’s QBIC system [38], several shape features are utilized, including area, eccentricity, circularity, major axis of inertia and higher-order algebraic moment invariants. 

All these shape features are combined into one feature vector, and shape similarity is measured using a weighted Euclidean distance metric. However, the shape-based search used in this system cannot differentiate between noise and a class-preserving deformation. Furthermore, it is challenging to objectively validate similarity measures used for indexing, because intuitive similarity (as perceived by the system designers) may not correspond well with meaningful similarity in the database. In formulating anatomic shape representations, we need to simultaneously ensure that the generated shape descriptions can be efficiently recovered and employed in shape alignment, recognition and comparison.

In this section, the technical requirements for the database engine were addressed. For images and descriptors storage, the 2016 Microsoft SQL Server engine with In-Memory OLTP (On-line Transaction Processing) component has been used. The following technical requirements were taken into consideration: efficient data processing, fast return response, minimum execution time of queries, efficient CPU usage and small log files.

Optimized tables store data using multiple versions of data from every row—this technique is characterized by non-blocking multi-version optimistic concurrency control and by lock exclusion for obtaining better performances in contrast with common tables. The advantages of using this type of table are the following:

➢ the table rows are read and written in the main memory;
➢ the entire table resides in the main memory;
➢ there is an option for stable data;
➢ if the option above is checked, a copy of the table will be stored on disk;
➢ data stored on the disk is only used at recovery;
➢ this type of table is interoperable with tables saved on disk.

Moreover, these tables can also be accessed by natively compiled stored procedures and saved on disk. Using these stored procedures may improve performance, because at compile time they are very well optimized and they only interact with memory saved tables.

There are several indexes that can be used with this type of table, including optimized indexes for in-memory OLTP such as hash index or non-clustered index, while common indexes can also be used. Custom indexes for in-memory OLTP only exist in memory, they do not persist on disk and every operation on these indexes is not logged in the log file. Their structure is constructed when the table is also created or when the server where the database resides is started.

A hash index is made up of a bucket collection organized in an array. There is a hash function that maps all index keys in the corresponding bucket with the hash index. The index characteristics are:

SQL Server only has one hash function that is used for all hash indexes;

➢ the hash function is a deterministic function;
➢ multiple indexes can be mapped in the same bucket.

Hash indexes are efficient with precise values searching queries, but the returned values are not sorted. Hash indexes are optimized for queries that use the equality operator and they support full index scans [39].

Non-clustered indexes offer everything that hash indexes do, plus range value search queries and sorted return values. The rows are returned according to the sorting option selected when creating the index. These indexes are unidirectional, since they only return the rows in the order established at the beginning [40]. One issue that must be considered when using such indexes is related to memory consumption. A hash index has a fixed dimension given by the number of buckets. For non-clustered indexes, the necessary memory resource is determined mainly by two parameters, namely: the number of rows and the dimension of columns which store index key.

Every table must have at least one index. It is necessary that one column with PRIMARY KEY constraint exists, which automatically creates an index. A column from an optimized in-memory table can be part of a hash index, but, also, from a non-clustered index. An optimized table could have a maximum number of eight indexes, including the PRIMARY KEY index. Also, the indexes must be created at the same time as the creation of the table and it is recommended that these indexes be associated only with intensively used columns (the garbage collections work well when the indexes are frequently used, otherwise it will not function optimally for the version of old rows). Indexes can be applied over non-null columns and they support BIN2 collations.

The requirements for using memory-optimized tables are:

➢ installing a 2016 SQL Server on 64-bit servers with NTFS file system;
➢ the free storage space must be twice the size of the table;
➢ the processor should support CMPXCHG16B instructions;
➢ the recommended maximum size for this type of table is 2TB;
➢ RAM memory should be at least twice the size of the table.

In order to store matrix or array values, it is recommended to use the JSON format. SQL Server 2016 offers functions for processing JSON format texts. Information in JSON format can be stored as text in a standard column in SQL Server, and for returning certain values from the JSON object; predefined SQL functions can also be used.

### 2.3. Object Tracking

Object tracking is a thriving research field in computer vision which is applied in video surveillance, traffic control, military intelligence as well as medical domains [41]. In our study, we showed an adapted version of the mean-shift process for tracking telocytes. We defined the geometry of the chosen kernel and proposed pre-processing techniques in order to increase the image contrast, thus enhancing the cells’ tracking. However, tracking migrating cells is a real challenge: each cell is different, having its own configuration, meaning that we needed to adapt current algorithms to new tasks. One important factor in automatic tracking is the acquisition modality. In our paper, we considered phase contrast microscopy.

**Figure 3:**
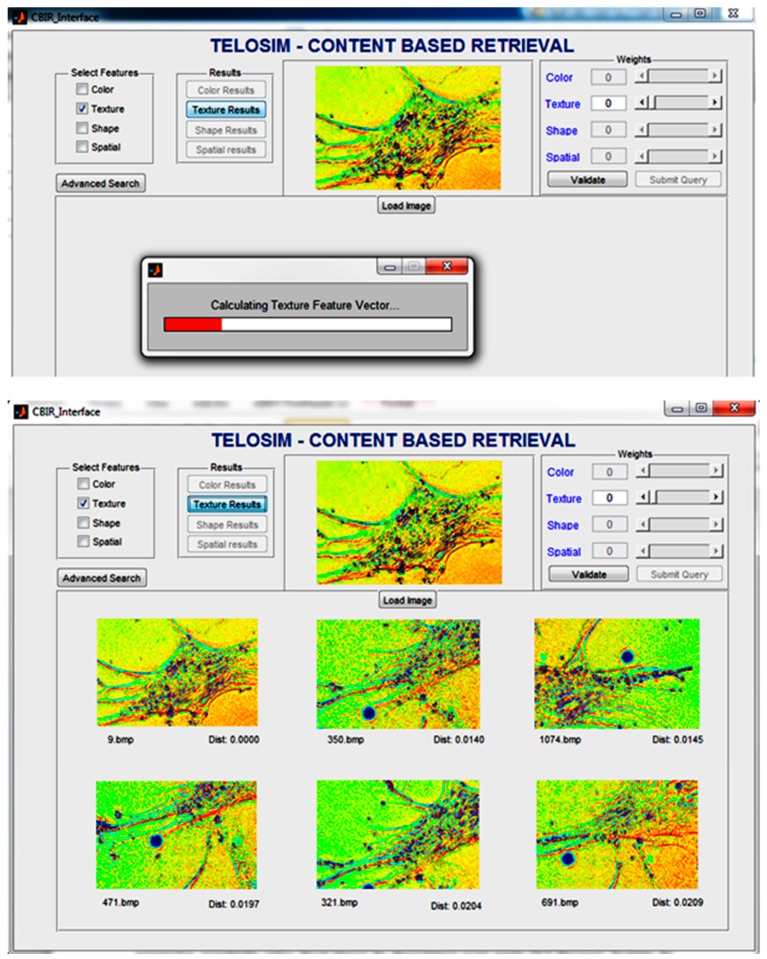
Example of similar images based on texture features. First, the texture feature vector is computed on the query image and then it is compared with vectors from the database.

The two main approaches for cell tracking are image segmentation and model adjustment. Image segmentation implies detecting the cells through a set of specific properties, such as color, shape, texture, etc. Using a CBIR system based on these features, one can choose a certain property or a weighted combination of them and search for images (or parts of an image) that are similar to the query image in terms of a chosen distance (for example Euclidean or Mahalanobis distance). An example of such a matching is illustrated in Figure 3.

In the first frame, the aimed objects (in our case, the cells) were detected based on a set of properties. Then the tracking was performed by object pairing between frames. This approach works fine when the cells are well defined, but it fails when the borders overlap or when the set of properties cannot be entirely visualized as a result of acquisition modality.

Model adjustment implies tracking a certain shape. This approach doesn’t track all objects in a video sequence, but only specific ones. In the case of cells tracking, we established a certain configuration (e.g., black spot surrounded by a white halo) which was tracked from one frame to another.

In this paper, the mean shift algorithm used for tracking a general type of cells was adapted [42]. The mean shift process is utilized for tracking spatial changes in color configuration. Since phase contrast microscopy was used for image acquisition, we modified the algorithm so that it would only support gray-level images. The mean shift process is meant to find where, in a given perimeter, the most pixels of a certain value are. The algorithm needs the shape of the perimeter where it searches for pixels with the specified value. Since a complicated geometrical form would be difficult to implement, the perimeter only depends on two factors: a center and a radius. The algorithm calculates a weighted average based on the position and intensity of the pixels. Let us consider a kernel *K* centered on *x* and radius *r* defined as follows:

$$K\left(x\right)=\{\begin{array}{c} 1,\;x\leq r\\ 0,\;x>r \end{array}$$

The mean for a set of gray-level pixel denoted with *S* is defined as:

$$m\left(x\right)=\frac{\sum_{s\in S}K\left(s-x\right)*s}{\sum_{s\in S}K\left(s-x\right)}$$

The mean-shift process replaces the center of the kernel *x* with the mean of it. The difference, *x*-*m*(*x*), indicates where most of the pixels are concentrated. This mechanism can be used to find either the brightest, the darkest or any other area in between. To adapt the mean-shift process to find the brightest zone, we defined the following mean:

$$m\left(x\right)=\frac{\sum_{p}K\left(p-x\right)*g\left(p\right)*p}{\sum_{p}K\left(p-x\right)*g\left(p\right)}$$

The position *p* is a 2-coordinate point that can be viewed as a vector in an actual implementation, while *g*(*p*) is the gray level of the pixel from the p position. To track the darkest pixel, we can use the same mean but with the gray level reversed (i.e., gp←(255-gp). We can use the mean-shift algorithm to find a certain pattern of gray level. For example, if we want to find the border between a bright area and a dark area, we can join two kernels. The first kernel is attracted by the bright pixels and the second one by the dark pixels. If *m*_1_(*x*) is the mean of the first kernel and *m*_2_(*x*) is the mean of the second one, we can define the mean for finding the border as follows:

$$m\left(x\right)=\frac{m_{1}\left(x\right)+m_{2}\left(x\right)}{2}$$

Of course, the previous equation can be generalized to shift the border closest to the bright or dark area. To do that, the weights, *w*_1_ and *w*_2_, of the two means were changed. The equation therefore becomes:

$$m\left(x\right)=\frac{w_{1}*m_{1}\left(x\right)+w_{2}*m_{2}\left(x\right)}{\left(w_{1}+w_{2}\right)}$$

**Figure 4:**
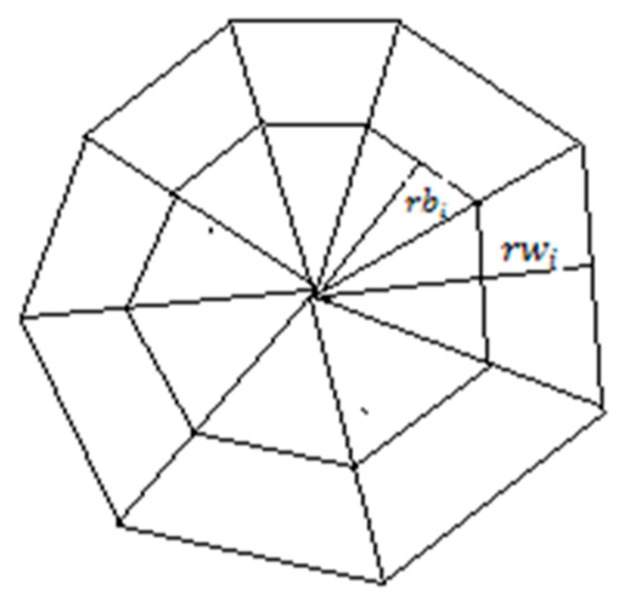
Two kernels centered in the same point. The inner kernel with the radius $rb_{i}$ is tracking the black area and the outer kernel with the radius $rw_{i}$ is tracking the white area.

This generalized form for tracking telocytes using the pattern of dark area surrounded by a white halo was used. Next, the geometry of the kernel and the mathematics that define it was presented. We assume that the kernel was centered at the position (x0, y0). To simplify things, we made all calculations in an orthogonal system centered at (0, 0) and then transposed the system at (x0, y0). In order to track a dark area surrounded by a white halo, we used two kernels centered in the origin of the orthogonal system. Each kernel was divided into eight parts, as illustrated in Figure 4. Splitting the kernel enabled the tracking to support different shape variations in cell morphology.

**Figure 5:**
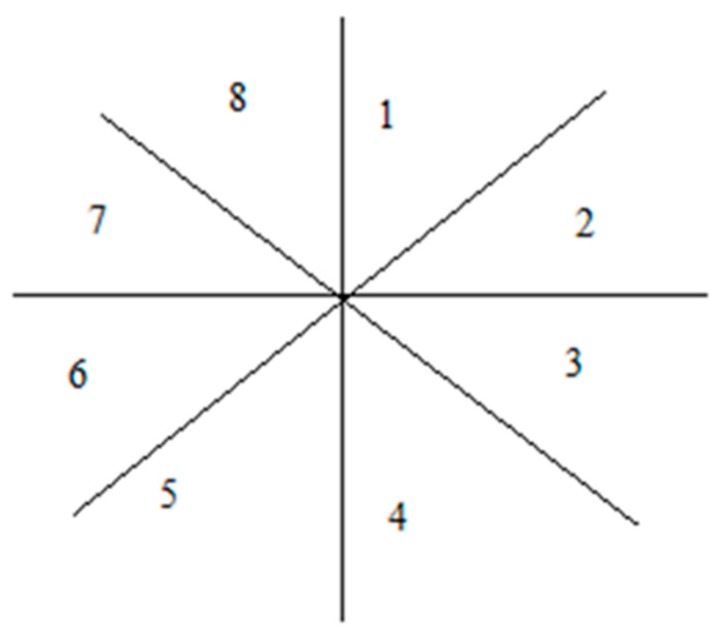
Kernel parts enumeration.

The white kernel had the radius denoted with $rw_{i}$ and the black kernel had the radius noted as $rb_{i}$. We considered $\;rb_{i}=rw_{i}*f,\;0<f<1$. To resist deformations in morphology, each of the eight radiuses was updated after each running of the algorithm. One problem was determining the geometrical place of the points in each kernel. We counted each part of the kernel as indicated in Figure 5.

**Figure 6:**
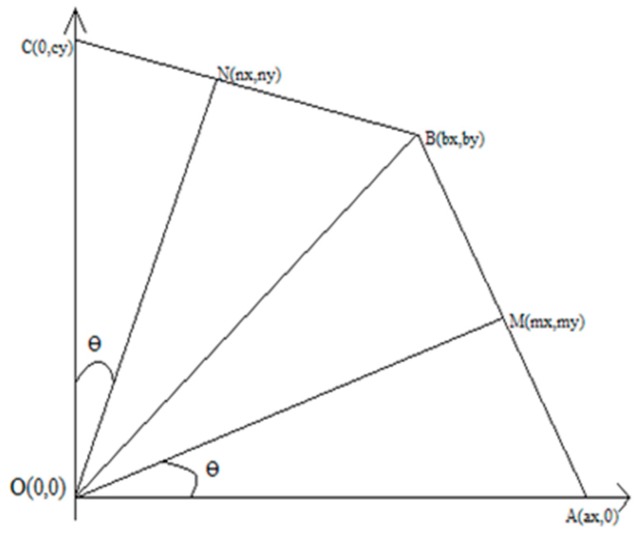
The length of the $\bar{\mathrm{ON}}$ segment is ${\mathrm{rw}}_{1}$ and the length of the $\bar{\mathrm{OM}}$ segment is **rw_2_**.

In order to determine the coordinates of each triangle that represented a part of the kernel, it was sufficient to calculate coordinates for triangles 1 and 2 and then use reflections in the Ox and Oy axes for the rest. In Figure 6 we extracted triangles 1 and 2 and positioned them into the axes system.

Since there were eight parts, the angle became $\theta =\frac{\pi }{8}$.

$$\frac{nx}{rw_{1}}=\cos\left(\frac{\pi }{2}-\theta \right)=\sin\left(\theta \right)=>nx=rw_{1}*\sin\left(\theta \right)$$

$$\frac{ny}{rw_{1}}=\sin\left(\frac{\pi }{2}-\theta \right)=\cos\left(\theta \right)=>ny=rw_{1}*\cos\left(\theta \right)$$

$$\frac{rw_{1}}{cy}=\cos\left(\theta \right)=>cy=\frac{rw_{1}}{\cos\left(\theta \right)}$$

Since *N* is the middle of BC, it results:

$$nx=\frac{bx}{2}=>bx=2*nx$$

$$\frac{by+cy}{2}=ny=>by=2*ny-cy$$

We have determined the coordinates of the triangle OBC and, similarly, the coordinates of point A, meaning:

$$\frac{rw_{2}}{ax}=\cos\left(\theta \right)=>ax=\frac{rw_{2}}{\cos\left(\theta \right)}$$

We found the coordinates of triangles 1 and 2. In order to determine the coordinates for the rest of the triangles, we used reflections between the Ox and Oy axes, thus delimiting the perimeters where the points from the kernel were. To find out whether a point P(x,y) is a triangle ABC, we drew a line parallel to Ox from P to the right and counted how many times the drawn line intersected the sides of the triangle. If this number was odd, then the point fell within the triangle. To determine the number of intersections, we equalized the equations of the drawn line with the equations of the sides of the triangle. In order to find out what points were in a triangle (i.e., in one of the eight parts of the kernel), we had to iterate all points in the image. That is an approach to brute force that does not succeed in large images. Instead of iterating through all the pixels, we established a rectangle in which we framed the entire kernel. The upper left corner of the rectangle had the coordinates (x0-*rw_i_*, y0+max(*rw_i_*)) and the lower right corner, (x0+*rw_i_*, y0-*rw_i_*), where *rw_i_* was the maximum radius of one part of the kernel and (x0, y0) was the center of the kernel.

This drastically reduced the complexity, because the mean-shift algorithm is a local algorithm, so the radius *rw_i_* cannot be very high. In order to adapt *rw_i_*, we determined the weight center of each triangle. In this way, we ensured that the radius was large enough to include the tracked zone or, on the contrary, that it was not too large. For example, if the shape of a cell has changed and the white zone became closer to the kernel’s center, the radius needed to be reduced. To detect the change, we calculated the weight center for each triangle and we detected whether the zone had reduced or increased. The exact relation was:

$$rw_{i}\left(t+1\right)=k*\left(\beta \bar{d\left(t\right)}+\left(1-\beta \right)*d_{i}\left(t\right)\right)$$

where $rw_{i}\left(t+1\right)$ was the radius at (*t* + 1), $rw_{i}\left(t\right)$ was the radius at the moment *t*, $d_{i}\left(t\right)$ was the distance from the weight center to the center of the kernel, $\bar{d\left(t\right)}$ was the mean of all the $d_{i}\left(t\right)$ terms and β and k were constants that depended on image quality and were established at implementation.

Due to the fact that images were acquired by phase-contrast microscopy, the white and black areas were not very easily distinguishable. For this reason, we used the histogram equalization technique to enhance the contrast and then we binarized the image using a threshold that depends on the quality of the image representing the tracked cell. The threshold is not a general feature of the whole image but a local characteristic that depends on the contrast of the observed cell.

**Figure 7:**
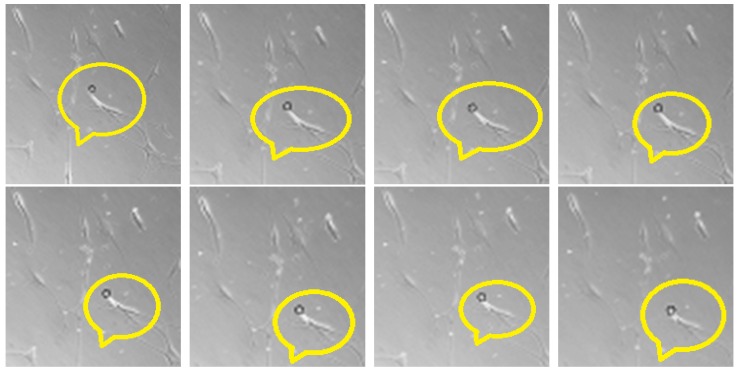
The black circle (inside the emphasized region) represents the center of the kernel that tracks the white cell. The order of frames is from left to right and from top to bottom. It is seen that the cell is shrinking with each frame and the kernel converges to the center of the cell.

Since we had to look at a dark zone surrounded by a white halo, if the image of the cell was well contrasted, then the threshold might have been lower compared to the case when the image was not so well contrasted. On the set of images chosen for testing, the threshold was 200. To better show the convergence, we chose an initial point situated near the actual cell. As it can be seen in Figure 7, the algorithm converged to the center of the cell. We used an initial radius of 16 and the constants k=1.55 and β=0.05.

We structured the main outcomes of the modeling component of this study as follows:

○ development of mathematical modeling and constitutive characterization of telocytes based on available experimental data. Study of the viscoelastic and hyperelastic constitutive laws for modelling telocyte behavior. The influence of the micro- and nano-scale on computational approaches;
○ the assessment of the biological and electromechanical framework of the problem;
○ model construction (geometry generation), identification of the constitutive law (material property), loading and boundary conditions;
○ validation of the finite component type;
○ numerical reproduction for the interpretation of telocytes behavior. Parametric studies concerning the assessment of the material constitutive parameters and changes during different charges and boundary conditions;
○ using the database of available experimental sources to validate the finite element model. The potential developments in finite element modeling of telocytes behavior were discussed;
○ theoretical study to identify the statistical properties of telocytes that could be used as features for their classification, detection and tracking in medical videos;
○ integration of various telocyte images into a multimedia database; establishing specific methods for indexing and efficient content-based searching for similar images;
○ software application for detection and recognition of telocytes in medical images;
○ software application for tracking and annotating telocytes in digital medical videos;
○ e-learning content that presents annotation, their roles and functionality, as resulted from the current paper, as well as from the group’s previous research.

Modeling the living cell behavior has always been a difficult task, even for cell types more common than telocytes. In this regard, there are scientific and technical barriers, because live cell imaging is difficult to carry out. Creating models implies chemical, mechanical, biological and often electrical approaches. Thus, a multidisciplinary approach is needed for a better understanding of the behavior of living cells.

For instance, Lim et al. [43] analyzed several mechanical patterns that have been developed to define mechanical responses of living cells when exposed to both short-term and longer-acting forces. Mechanical models included cortical models with a fluid core, generally used for suspended cells, solid patterns, widely applied for adherent cells, power-law structural damping model, more suitable for analyzing the dynamic nature of adherent cells, and, lastly, the biphasic model that has been broadly applied to view musculoskeletal cell mechanics. According to these models, once these factors have been appropriately addressed, future experiments can be carried out in order to determine more precise and specific mechanical types of living cells, that distinguish themselves due to architectural heterogeneity, relations for each of their unique subcellular sites and parts, and active forces that are taking place inside the cells. More authentic mechanical types of living cells can also contribute to the research of telocytes whose particular aspect plays a key role in their modeling.

Having in sight the possibility of knowledge transfer for further biological and bio-medical applications, the main issues which have been analyzed in this study are structured into three main research areas, as follows: computer assisted training, integrated telemedicine systems and the use of information systems in telocyte research [37,44].

#### 2.3.1. Computer-Assisted Training

○ analysis of current research in e-learning, distance learning and computer-assisted training;
○ identification of the positive and negative aspects regarding the use of multimedia information systems in the educational process;
○ analysis of existing software systems from the point of view of their use in online training;
○ studying current standards for designing graphical user interfaces for remote training systems;
○ analysis and implementation of current international standards in an e-learning platform of its own design, that allows participants to gain access to modern knowledge and forms of assessment;
○ elaboration of multimedia educational materials for professional training and promotion of the use of new technologies for the health sector personnel.

#### 2.3.2. Telemedicine Integrated Systems

○ development of a communication system with people from the health academic environment;
○ identifying efficient technical solutions to develop scalable multimedia architectures for complex health teleservices;
○ database design aimed at integrating information pertaining to the telemedicine system.

#### 2.3.3. Informatics Systems for the Study of TCs Behavior

○ development of an informatics system like a virtual learning environment platform that integrates a complex database, accessible through a web interface;
○ design of user interfaces for the informatics system (students, teachers, administrators, content creators, etc.);
○ methods for users and access management based on users’ rights; methods for content management: creation, storage, approval, distribution and publishing.

## 3. Material and Methods

Our research strategy is developed in several directions in order to study the potential functional differences between the cell body and Tps.

### 3.1. Human Samples and Ethics Statement

Human myometrium biopsies were collected from pregnant and non-pregnant women (n = 8, in each group). All tissue samples were obtained in accordance with protocol no. 12290 (18 August 2017) approved by the Ethics Committee of Alessandrescu-Rusescu National Institute of Mother and Child Health in Bucharest, Romania. All patients donating uterine tissue samples that were included in the study had signed a written informed consent. Subjects were not taking regular medication for chronic illnesses. Strips of non-pregnant myometrium were collected from hysterectomy specimens (for benign conditions) of premenopausal women, from the supraisthmic region. Small strips of pregnant myometrium (38 to 40 weeks of gestation) were taken during C-section from the upper margin (in the midline) of the lower segment transverse incision.

### 3.2. Myometrial Cell Cultures

Samples of human myometrium were collected into sterile tubes with Dulbecco’s Modified Eagle’s Medium (DMEM), supplemented with fetal bovine serum (FBS) 2%, HEPES (1.5 mM), penicillin (200 IU/mL), streptomycin (200 UI/mL) and amphotericin B (Fungizone, 0.50 µg/mL) (all from Gibco/Life Technologies Ltd., Paisley, UK). The tubes were transported on ice to the cell culture laboratory, where the myometrial samples were sliced under a stereomicroscope (Nikon Instruments Europe BV, Amsterdam, The Netherlands) in fragments of approximately 1 mm^3^, then washed and incubated with gentle agitation for 30 min, at 37 °C, with collagenase Ia, 10 mg/mL, and DNAase I, 0.1 nm/mg (both from Sigma-Aldrich, St. Louis, MO, USA) in DMEM supplemented with FBS 10%, HEPES 1.5 mM, 100 IU/mL penicillin, 100 UI/mL streptomycin and 0.25 µg/mL fungizone (Gibco/Life Technologies Ltd., Paisley, UK). The protocol was described in detail elsewhere [45].

Telocytes were investigated and identified in a myometrial interstitial cell culture based on morphological criteria and prepared for time-lapse video microscopy by being suspended with a density of 5 × 103 cells/mL in DMEM culture medium, supplemented with 10 % FBS and 100 U/mL penicillin—100 μg/mL streptomycin and were maintained in 37 °C and 5% CO_2_ humidified environment. Cell cultures were passaged every 2 days at 90% confluence and detached by trypsinization. The cell suspension was centrifuged at 1200 rpm for 3 min at room temperature. The supernatant was removed and fresh medium was added to resuspend the cells. An amount of 20% of the cell suspension was transferred into a new flask for continued culture. The cell density was measured using a hemocytometer and adjusted to between 5 × 10^5^ and 1 × 10^6^ cells/mL. For image and time-lapse acquisition, the suspension was plated in standard 6-well plates and placed under the microscope (Zeiss Observer 200 microscope equipped with stage micro incubator, Axiovision software package, Carl Zeiss, Jena, Germany). The acquisition was performed using either a 63× LD-Plan-Neofluar objective (for detailed images) or a 10× A-Plan objective (for large field time-lapse recordings). All recordings were performed at 37 °C and 5% CO_2_ humidified environment.

### 3.3. Cells Were Studied as Follows

The cellular patterns of cultured telocytes were evaluated using fluorescence microscopy techniques. In addition, cellular studies of membrane fluidity, transmembrane electrical potential and generalized polarization of fluorescence were performed. These studies provided significant information regarding the physical behavior of cellular membranes while aiming to highlight the behavioral differences between the cell body and telopodes.

Image acquisition—the growth patterns of telocytes were recorded in specific culture-appropriate conditions in Petri dishes for various intervals. The images and videos were used to create a database aimed at providing the necessary elements for the physical characterization of telocytes. The cells were grown in Petri plates and visualized with a Nikon inverted TE200 microscope attached to the incubation hood, using objectives with a wide field, 4X and 10X (Nikon Instruments Europe BV, Amsterdam, The Netherlands).

Electron microscopy—after fixation, small tissue samples (about 1 mm^3^) of human pregnant and non-pregnant myometrium were embedded in epoxy resin using a protocol previously described [9]. Ultrathin sections were mounted on 50-mesh grids and double stained with uranyl acetate and lead citrate. The grids were examined using a Phillips 301 electron microscope or a CM 12 Philips electron microscope (Philips Research, Eindhoven, The Netherlands) at an acceleration voltage of 60 kV. Images were captured using an Olympus Morada CCD camera, 16 bits, 11 megapixels (Olympus Soft Imaging Solutions, Münster, Germany). We also aimed to correlate the distribution, localization and activity of mitochondria focusing on potential functional differences between various cellular segments. All these data would later become input parameters for the mathematical modeling of telocytes’ behavior.

Mathematical modeling—both numerical and analytical/semi-analytical approaches to the behavior modelling of living cells are difficult tasks, mainly due to the fact that material parameters of living biological tissues are submitted to set and laboratory measurements that are difficult to obtain. Based on the in vitro behavior and appearance of telopodes, an appropriate semi-analytical model for their characterization has been proposed, using viscoelastic elongation [43,46]. For the numerical model, we used the Ansys software (ANSYS, Inc., Pittsburgh, PA, USA) for multiphasic viscoelastic model with the same conditions as in the semi-analytical model in order to compare the two solutions.

The objectives of the mathematical and numerical modeling of telocytes behavior were the following:

○ Theoretical modeling and numerical simulations concerning telocyte behavior were aimed at identifying certain constitutive properties and achieving a description of their structure in order to elucidate their role in regenerative medicine. The required chemical, biological, electrical and mechanical aspects were taken into account for appropriate formulation;
○ Telocyte behavior analysis from an electro-mechanical point of view revealed important information on heterogeneity, stiffness and strength of telocyte structure in comparison with other biological materials;
○ The development of a finite element method (FEM) framework for the study of telocyte behavior regarding the mechanical response to various types of stimuli;
○ Validation of the numerical model in order to ensure that quantitative and qualitative data matches that of laboratory tests;
○ The processing of modeling results is to be integrated into the general software package concerning the TCs presentation.

**Figure 8:**
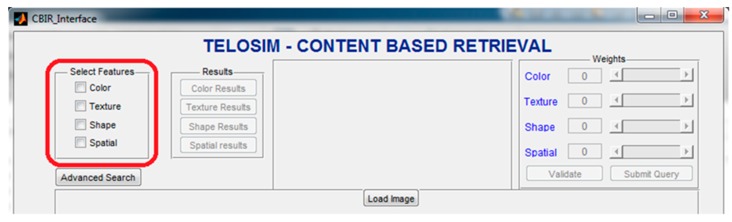
Content Based Image Retrieval (CBIR) Application.

The content-based image retrieval (CBIR) application, image processing and object tracking are illustrated in Figure 8.

The three-dimensional reconstruction of an object can be seen as the reproduction of a depth-map. Given sequences of images as input, the output consists of the distances of each point of the target object from a reference point. After reconstruction, the object can easily be visualized with a computer graphic application. Using such a model-based software could be useful not only in facilitating a correct diagnosis, but also for future educational purposes.

Telocytes behavior could be understood using the ultimate image processing techniques that have been created in order to characterize and describe the following topics:

➢ detection and recognition of telocytes within a digital image;
➢ setting up an image database with efficient indexing and CBIR;
➢ tracking of telocytes within digital medical videos based on a previous detection.

In our study, already available medical videos have been used in order to depict telocytes in vitro. Furthermore, we proposed an adapted version of the general tracking scheme. The main principle of tracking algorithms of a current object is represented by the considerable difference between non-aligned and aligned subjects. However, this aspect is not guaranteed in biological imaging. Our study therefore also focuses on identifying factors that may affect the tracking accuracy in the biological domain.

After measuring the trajectories of telocytes, raw video data was used in order to obtain feature measurements of TCs migration. Great potential in the study of TCs migration could be revealed from fully-automated cell video analysis, feature selection as well as statistical tests for all the data in the current study. Tracking analysis and the TCs feature measurement helped us obtain a numerical estimation of cells migration. The significant changes in the behavior of TCs could be determined automatically using a combination of tools like feature selection and statistical tests.

e-Learning platform design (Sharable Content Object Reference Model—SCORM) nowadays constitutes the advanced direction of more traditional learning methods. It is currently considered that an education technology has two main directions: consolidating the learning management system (LMS) and the development of reusable content, the latter being the main target of our research study. The picture archiving and communication system (PACS) is the most used medical-based image information system. These systems use HL7 and DICOM forms for information and images. Our proposal for content reusability is to use SCORM conformant. It is also planned to establish an authoring tool which will be used to transform information into SCORM courses from DICOM images [47]. SCORM specifications are used by e-Learning technologies for most of the courses developed today. Using the same version of SCORM with minimum adaptation of any other SCORM-conformant learning management system enables the upload of both the course and its components, while also permitting the interchange of lessons among courses. Experimental possibilities could be elaborated using new materials and SCOs (Sharable Content Objects) created by various institutions, and could be mixed to obtain a new course that would be restructured and packaged entirely in order become a unique entity. The opportunities to design unique courseware that can be customized according to specific learning needs are created by the ability of disparate knowledge objects to work together in unanticipated ways. Both simple text-based pages and interactive media content with various levels of complexity are supported by SCORM.

E-Learning using SCORM has the following representative properties:

➢ accessibility (any location is supported);
➢ interoperability (it can be used by different e-learning instructional platforms);
➢ durability (it keeps up with technological advances without the need for major redesign);
➢ reusability (it can be used by different applications, platforms or tools);
➢ cost efficiency and facile maintainability.

As one of the first theoretical models of telocytes, it should be regarded as very helpful for prospective reference, since telocytes are a new area of study, with much more research being required in order to gain a deeper understanding of all the processes they are involved in.

## 4. Conclusions

The general purpose of this paper was to gain a deeper understanding of the relationship between telocytes and their mechanical/structural properties, which play a key role in determining their functions. This paper provides new insights regarding the morpho-functional characteristics of telocytes by proposing experimental models based on monitoring cellular activity both in situ and in vitro. Experimental information was used to develop mathematical and numerical methods for the analysis of the physical texture of telocytes. Mathematical analysis revealed important information about the homogeneity, hardness and resistance of telocyte structure in comparison to other types of biological materials (culture medium and other cell types). Cellular activity models were monitored in vitro, therefore supporting the creation of databases of telocyte images. The obtained images were analyzed using segmentation techniques and mathematical models in conjunction with computer simulation, in order to depict telocytes behavior in relation to surrounding cells. This paper brings an important contribution to the development of bioinformatics systems by creating software-based telocyte models that could be used both for diagnostic and educational purposes. These type of advances in both biomedical and mathematics and computing fields will aid the dissemination of research findings and cooperation between professionals from different scientific fields.

Telocytes might play an even more important role in the future, offering the possibility of regenerating various human tissues. At the same time, understanding their behavior in cell cultures influenced by the surrounding conditions (neighboring cells, medium properties) could lead to applications to be developed in the near future. New methods must be developed in order to replicate the highly specialized structures of TCs in vitro.

Regarding the mathematical modeling, the first step that we performed was proposing semi-analytical and numerical models for the viscoelastic elongation of the telopodes, since they are specific to telocytes. However, for a deeper understanding of telocytes’ biomechanics and physiology, we must develop further models in alignment with better laboratory tests. Experiments have shown that a more accurate biomechanical model of the behavior of living cells can be obtained by including, for example, the specific properties of each subcellular component, as well as the forces acting within the cell.

As for the e-learning system, the main theoretical and practical contributions remain:

○ the theoretical study regarding the standardization, organization and delivery of educational content using e-learning systems;
○ the elaboration of criteria for the use of e-learning systems in both academic and healthcare environments;
○ the production of multimedia educational materials meant to assist the training, testing and evaluation of knowledge for students and professionals in the health sector.

This paper promoted an interdisciplinary approach which took place through a public–private partnership and resulted in the production of an efficient software package that allowed the simulation and modeling of the behavior of telocytes within the tissular processes of signaling and regeneration. Since biological, chemical, electrical and mechanical aspects were involved, a multidisciplinary approach was needed in order to achieve a better grasp of living cells’ behavior.

Although quite a few technical barriers persist, understanding telocytes’ involvement in cell signaling and tissue regeneration could lead to an unexpected opportunity for developing future treatments. Future strategies will undoubtedly focus their efforts in elucidating the complex roles of telocytes taking place in close proximity to the surrounding cells, which they influence via the exosomes or shedding microvesicles that they released. The successful establishment of a realistic models by which telocytes respond to surrounding tissue matrices increases the number of possible applications of these cells in biological and medical research.
